# Physical Resilience and Its Influencing Factors Among Older Patients with Fragility Fractures: A Cross-Sectional Study Based on Latent Profile Analysis

**DOI:** 10.3390/healthcare14131923

**Published:** 2026-07-01

**Authors:** Jing Chen, Wanqi Li, Jiale Liu, Chun Shen, Yi Jiang, Jiao Hua

**Affiliations:** 1 Department of Nursing, Affiliated Hospital of Jiangnan University, Wuxi 214122, China; cj02303@163.com (J.C.); liwanqi0427@163.com (W.L.);; 2 Wuxi School of Medicine, Jiangnan University, Wuxi 214122, China; lacey616@163.com (J.L.); 6242850005@stu.jiangnan.edu.cn (C.S.)

**Keywords:** physical resilience, fragility fracture, latent profile analysis

## Abstract

**Background:** Physical resilience (PR) plays a critical role in the functional recovery of older adults following fragility fractures, yet individual heterogeneity remains underexplored. Objective: To identify latent profiles of PR among older adults following fragility fractures and to examine their biopsychosocial predictors. **Methods:** From October 2025 to March 2026, 224 older adults with fragility fractures were recruited using convenience sampling from a tertiary hospital in China. Data were collected using demographic questionnaires, the Physical Resilience Instrument for Older Adults, Perceived Social Support Scale, and General Self-Efficacy Scale. Latent profile analysis identified profiles, followed by multinomial logistic regression examining biopsychosocial predictors. **Results:** Three distinct resilience profiles emerged: low resilience—physically and mentally vulnerable (31.7%); moderate resilience—limited adaptation (51.5%); and high resilience—potential activated (16.8%). Significant profile predictors included handgrip strength, nutritional risk, marital status, general self-efficacy, and perceived social support (*p* < 0.05). **Conclusions:** The distinct heterogeneity of PR among older adults with fragility fractures underscores the necessity for tailored, risk-stratified nursing. In clinical practice, interventions for the highly vulnerable low-resilience group should prioritize multidisciplinary nutritional optimization and early physical rehabilitation. For patients with moderate resilience, integrating spousal support and cognitive-behavioral strategies is crucial to enhance self-efficacy and prevent functional decline. For the high-resilience cohort, leveraging robust social support networks and empowerment-based strategies can maximize their intrinsic recovery potential.

## 1. Introduction

Fragility fractures, a severe complication of osteoporosis, have emerged as a major public health challenge for the global aging population [1]. Statistically, approximately 200 million individuals worldwide are affected by osteoporosis, resulting in an estimated 8.9 million osteoporotic fractures annually—equivalent to one fracture every three seconds [2]. In China, the prevalence among individuals aged 60 and older reaches as high as 24.7% [3]. Critically, the mortality and disability rates associated with fragility fractures (particularly hip fractures) are exceptionally high; the one-year mortality rate following a fracture can reach 36%, and approximately 50% of survivors are left with varying degrees of functional impairment or disability [4]. Such acute trauma frequently accelerates physical decline in physical function among older adults, potentially triggering long-term severe disability [5,6], thereby imposing a profound burden on both families and healthcare systems.

In orthopedic rehabilitation and geriatric care, despite the implementation of identical standardized clinical pathways or complication prevention protocols for fractures of similar severity, ultimate functional recovery outcomes have exhibited significant heterogeneity. Some patients experience persistent functional decline, whereas others manage to achieve optimal functional recovery [7]. This heterogeneity indicates that a patient’s final recovery trajectory is determined not solely by the severity of the trauma, but also relies heavily on the intrinsic potential and physiological reserves the individual can mobilize when adapting to acute stress.

Physical resilience (PR), defined as an individual’s capacity to resist functional decline, recover, or optimize existing physical function following an acute health stressor, serves as a key concept underlying this perspective [8]. Distinct from the traditional deficit-accumulation concept of frailty, PR focuses on an individual’s reserves and stress-resistance capacity. It not only predicts patients’ clinical outcomes but is also considered a core element in achieving healthy aging [9]. The conceptual model proposed by Whitson et al. explicitly posits that PR is a specific health outcome jointly driven by multiple antecedent factors—including an individual’s underlying physiological reserves, psychological resources, and social environment—under the influence of acute stressors or physiological challenges [10]. This multidimensional framework informed the selection of predictor variables in the present study. Previous studies have indicated that following severe stress, older adults with higher levels of PR demonstrate significantly better recovery of postoperative activities of daily living (ADL) and overall quality of life compared with their low-resilience counterparts [11]. Consequently, identifying the antecedent factors influencing PR has important implications for the early prediction of clinical outcomes and the formulation of targeted multidisciplinary interventions.

Current clinical and rehabilitation research in this field still has several limitations. First, contemporary nursing research on fragility fractures rarely includes in-depth analyses of how patients mobilize their intrinsic potential to drive functional recovery. Furthermore, previous evaluations of PR have predominantly relied on total scale scores or mean values, an approach that masks the high degree of heterogeneity within the older adult population. Latent profile analysis (LPA), as a person-centered statistical method, can effectively address these methodological limitations by objectively and accurately identifying phenotypes with distinct characteristic combinations within a population [12]. While latent profile analysis (LPA) and other person-centered approaches have been successfully applied to identify resilience or symptom trajectories in other clinical populations (e.g., oncology and chronic pain), their specific application to PR among older adults with fragility fractures remains largely unexplored. Therefore, this study aims to explore the latent profiles of PR among older adults with fragility fractures using LPA, analyzing the characteristics and antecedent predictors of different categories. We hypothesized that older patients with fragility fractures would exhibit distinct physical resilience profiles, and that membership in these latent profiles would be significantly predicted by specific sociodemographic characteristics, clinical variables, and psychosocial resources (e.g., social support and self-efficacy). Ultimately, this research seeks to enable the early identification of high-risk individuals on the verge of functional decompensation, thereby facilitating the development of tailored clinical and rehabilitation strategies.

## 2. Methods

### 2.1. Participant and Study Design

A cross-sectional study was conducted using convenience sampling to consecutively recruit 224 older adults with fragility fractures. Participants were enrolled from the orthopedic unit of a tertiary hospital in Jiangsu, China, between October 2025 and March 2026. All individuals provided written informed consent, with guaranteed data anonymity and the right to withdraw at any time. The study protocol was approved by the Ethics Committee of the Affiliated Hospital of Jiangnan University (Approval No. LS2025314) and complied with the 1964 Declaration of Helsinki. Informed consent was obtained from all participants (and their guardians) involved in this study. The study was approved by the Ethics Committee of the Affiliated Hospital of Jiangnan University.

The inclusion criteria were as follows: (1) Aged 60 years or older. (2) A confirmed diagnosis of a primary fragility fracture caused by low-energy trauma, consistent with the World Health Organization consensus [13]. Fractures were definitively diagnosed via standard medical imaging by attending orthopedic physicians, with acute vertebral fractures specifically requiring radiological evidence of acute compression. Consistent with clinical guidelines, such fractures inherently indicate severe osteoporosis; thus, prior bone mineral density (BMD) testing was not required. (3) Sufficient cognitive ability to provide voluntary informed consent.

The exclusion criteria for study participants were as follows: (1) fractures resulting from high-energy trauma (e.g., traffic accidents) or pathological fractures secondary to bone tumors or metastasis; (2) chronic inflammatory or metabolic bone diseases (e.g., severe rheumatoid arthritis) other than primary osteoporosis; (3) diagnosed psychiatric disorders or severe cognitive impairment (identified via pre-existing clinical diagnoses, such as dementia or Alzheimer’s disease, documented in the electronic medical records upon admission and confirmed by the attending physicians); (4) severe auditory or visual impairments precluding effective communication; and (5) severe dysfunction of vital organs (e.g., advanced cardiopulmonary, hepatic, or renal diseases) or terminal illnesses. The detailed patient screening and selection process is illustrated in a STROBE flow diagram (Supplementary Figure S1).

### 2.2. Sample Size

Sample size estimation was performed using G*Power 3.1.9.7. With a significance level of α = 0.05, a statistical power of 0.80, a conservative medium-to-small effect size (f2 = 0.12), and 12 independent predictors, the minimum required valid sample size was calculated as 156 participants. Considering a 20% expected rate of invalid questionnaires and data exclusion, the adjusted target recruitment sample size was 195 cases. Furthermore, the sample size requirement for Latent Profile Analysis (LPA) is not a fixed absolute threshold; rather, it is contingent upon the interplay among the total sample size, the number of indicators, and the expected number of profiles. According to methodological simulation studies by Tein et al., when a model anticipates large to very large profile separation, moderate sample sizes (e.g., *N* ≈ 250) are generally considered acceptable to yield reasonable statistical power for detecting latent classes and supporting stable parameter estimation [14]. While acknowledging that larger sample sizes are always optimal in finite mixture modeling, the 224 eligible older adults enrolled in this study were deemed sufficient to meet the fundamental statistical requirements for both latent profile grouping and subsequent predictive regression analysis.

### 2.3. Measurements

#### 2.3.1. Demographic and Clinical Characteristics

A demographic and clinical characteristics questionnaire was developed by the research team based on a literature review and expert discussion. Demographic data included age, gender, marital status, educational level, and average monthly household income. Clinical and health-related data comprised handgrip strength (HGS), Nutritional Risk Screening 2002 (NRS 2002) score, fracture site, and history of previous fractures.

Handgrip strength was measured using the EH101 digital hand dynamometer (Guangdong Xiangshan Weighing Apparatus Group Co., Ltd., Zhongshan, China) [15]. Given the severe pain and mobility restrictions associated with the acute phase of fragility fractures, the conventional seated posture was not feasible. Therefore, patients were assessed in a standardized, modified semi-recumbent position. While the Asian Working Group for Sarcopenia (AWGS) criteria are based on a seated position, previous clinical validation studies have demonstrated that handgrip strength measured in supine or semi-recumbent positions is highly correlated with seated measurements, making it a valid and reliable alternative for bedridden older adults [16]. To ensure reproducibility and high inter-rater reliability, all measurements were conducted by uniformly trained clinical researchers following a strict protocol. Specifically, the head of the bed was elevated to 30 degrees with the back supported by pillows to stabilize the trunk. A soft pillow was placed under the unaffected upper arm to maintain the shoulder joint at approximately 30 degrees of flexion, with the elbow joint extended as much as comfortably possible. Measurements were repeated three times for the unaffected hand with an interval of at least 30 s between trials, and the maximum value was recorded. Decreased handgrip strength was defined as <28 kg for males and <18 kg for females [17].

Nutritional risk was evaluated using the NRS 2002 [18]. A total score of <3 indicated no risk of malnutrition, whereas a score of ≥3 denoted a risk of malnutrition.

#### 2.3.2. Physical Resilience Instrument for Older Adults (PRIFOR)

Developed by Hu et al. [19] and cross-culturally adapted by Li et al. [20], this instrument is utilized to quantify the level of physical resilience in older adults following an acute health stressor. It comprises 16 items structured across three dimensions: positive thinking, coping and lifestyle adaptation, and belief and hope. Each item is rated on a 5-point Likert scale ranging from 1 (strongly disagree) to 5 (strongly agree), yielding a total score of 16 to 80, with higher scores reflecting greater resilience capacity. In the present study, the Cronbach’s α was 0.923, confirming the instrument’s robust reliability in this population.

#### 2.3.3. Perceived Social Support Scale (PSSS)

Originally developed by Zimet et al. [21] and translated into Chinese by Jiang [22], this 12-item tool encompasses three dimensions: family support, friend support, and other support. Responses are evaluated on a 7-point Likert scale from 1 (very strongly disagree) to 7 (very strongly agree), with the cumulative score ranging from 12 to 84; higher scores indicate stronger perceived social support. In our study, the scale exhibited good internal consistency, with a Cronbach’s α of 0.804. 

#### 2.3.4. General Self-Efficacy Scale (GSES)

Translated and revised by Wang et al. [23], this unidimensional scale is designed to assess an individual’s optimistic self-beliefs in coping with life’s difficulties. The scale contains 10 items, each rated on a 4-point scale from 1 (not at all true) to 4 (exactly true). The total score ranges from 10 to 40, where higher scores denote higher levels of self-efficacy. In this study, the Cronbach’s α coefficient for the GSES was 0.935.

### 2.4. Data Collection

Data collection was conducted using paper-based questionnaires administered by two uniformly trained investigators. Prior to the survey, standardized instructions were utilized to explain the study objectives and significance, with strict assurances provided regarding the confidentiality of participant responses and personal information. In instances where participants were unable to complete the questionnaires independently due to specific limitations, the investigators verbally administered the items and recorded the responses on their behalf. Upon completion, the questionnaires were collected on site and meticulously reviewed. Any inadvertently missed items were immediately clarified with participants. Questionnaires with unresolved missing data were excluded. Consequently, the final analyzed dataset was entirely complete, allowing for a complete-case analysis without requiring data imputation. To ensure the accuracy and integrity of the raw data, all data were independently double-entered and cross-checked by two researchers on the same day of data collection.

### 2.5. Statistical Analysis

Latent profile analysis (LPA) was performed using Mplus version 8.3 (Muthén & Muthén, Los Angeles, CA, USA) to identify distinct subgroups of PR by sequentially testing models with one to five profiles. To maintain conceptual purity and prevent confounding, LPA indicators were strictly limited to the sub-dimensions of Physical Resilience (PR). Psychosocial resources (e.g., social support, self-efficacy) were evaluated separately as independent predictors, ensuring a clear methodological distinction between the resilience phenotypes and their predictive factors. Model fit was evaluated using the Akaike information criterion (AIC), Bayesian information criterion (BIC), and sample size-adjusted BIC (aBIC), with lower values indicating a superior model fit. Classification accuracy was assessed via Entropy, where values closer to 1 indicate more precise classification, and an Entropy value ≥ 0.80 is generally considered to indicate clear class separation and acceptable classification precision. The Lo–Mendell–Rubin likelihood ratio test (LMRT) and the bootstrapped likelihood ratio test (BLRT) were utilized to compare neighboring models, where a significance level of *p* < 0.05 indicated that the *k*-profile model provided a significantly better fit than the (*k* − 1)-profile model. Furthermore, prior to analysis, we pre-specified two additional criteria for determining the optimal model: (1) a minimum class proportion of ≥5% to prevent the retention of spurious minor classes and (2) high profile classification stability, indicated by Average Posterior Probabilities (AvePP) ≥ 0.80 for all latent classes.

Descriptive and inferential statistical analyses were conducted using IBM SPSS Statistics 25.0(IBM Corp., Armonk, NY, USA). Continuous data with a normal distribution were expressed as mean and standard deviations (SDs), with two-group and multi-group comparisons analyzed using the independent-samples *t*-test and one-way analysis of variance (ANOVA), respectively. Non-normally distributed continuous data were presented as medians and interquartile ranges (IQRs) and were compared using the Mann–Whitney *U* test or the Kruskal–Wallis *H* test. Categorical and ordinal data were expressed as frequencies and percentages; group comparisons for nominal variables were performed using the Chi-square (*χ*^2^) test or Fisher’s exact test, whereas ordinal variables were evaluated using the Kruskal–Wallis *H* test. Finally, the optimal latent profile membership was utilized as the categorical dependent variable in a forced-entry multinomial logistic regression model. Guided by the theoretical framework of physical resilience—which posits that baseline physiological reserve and stressor severity inherently determine recovery trajectories—age, sex, comorbidity burden, and fracture site were prespecified as obligate clinical confounders. These variables were forced into the model regardless of their univariate *p*-values, alongside other variables demonstrating statistical significance (*p* < 0.05) in the preliminary analyses. Two-tailed values of *p* < 0.05 were considered statistically significant.

## 3. Results

### 3.1. Latent Profile Analysis of Physical Resilience

The overall physical resilience score for the 224 older adults with fragility fractures was 50.10 ± 8.12. In this study, the dimensions of PR among older adults with fragility fractures were used as observed variables to fit models 1–5, and the specific fitting indices of each model are shown in Table 1. As the number of extracted profiles increased, the values of AIC, BIC, and aBIC steadily decreased. Regarding classification precision, the 3-profile model yielded the highest Entropy value of 0.865, outperforming both the 2- and 4-profile models. Furthermore, the likelihood ratio tests (LMRT and BLRT) indicated that the 3-profile model provided a significantly better fit than the 2-profile solution (*p* < 0.001). Although the 4-profile model demonstrated a statistically significant LMRT value (*p* = 0.007) compared to the 3-profile model, it resulted in a decreased Entropy value (0.844) and the emergence of a splintered subgroup with a small sample proportion (10.3%). Taking into account the peak classification precision, statistical robustness, and the necessity to maintain parsimonious clinical interpretability while avoiding over-extraction, the 3-profile solution was ultimately selected as the optimal model. Crucially, this 3-profile model demonstrated exceptional classification stability. The Average Posterior Probabilities (AvePPs) for the three latent classes were 0.945, 0.932, and 0.940, respectively. All values substantially exceeded the pre-specified 0.80 threshold, indicating highly robust latent class assignment and minimal classification error.

**Figure 1:**
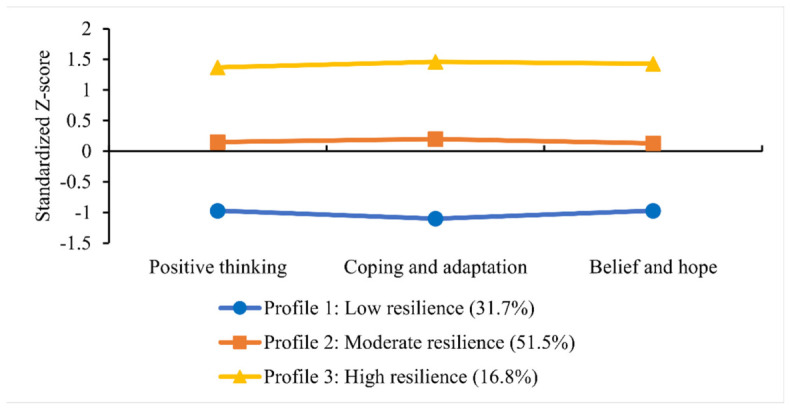
Latent profile characteristics of physical resilience among older adults with fragility fractures.

To account for the variations in the number of items and baseline scoring across the dimensions of PR, raw scores were transformed into standardized *Z*-scores (mean = 0, standard deviation = 1) prior to plotting the profile characteristics, thereby eliminating dimensional disparities and enabling cross-dimensional comparability. Consequently, the latent profile chart was plotted based on the conditional mean *Z*-scores of these dimensions (Figure 1). Based on the conditional mean *Z*-scores, the three distinct profiles were characterized and labeled as follows: Profile 1 (Low resilience—physically and mentally vulnerable): Comprising 70 patients (31.7%), this subgroup exhibited *Z*-scores below 0 across all dimensions, indicating comprehensive impairment. Profile 2 (Moderate resilience—limited adaptation): This was the largest subgroup, comprising 116 patients (51.5%), with scores hovering near the zero-line, reflecting the average sample level. Profile 3 (High resilience—potential activated): Comprising 38 patients (16.8%), this subgroup demonstrated *Z*-scores greater than 1 across all dimensions, manifesting a substantial advantage in resilience resources.

### 3.2. Baseline Characteristics and Univariate Analysis

The baseline characteristics and univariate analysis of variables across three physical resilience profiles are presented in Table 2. The univariate analysis revealed statistically significant differences across the three PR profiles with respect to age, marital status, educational level, per capita monthly household income, fracture site, history of previous fractures, handgrip strength, nutritional risk, general self-efficacy, and perceived social support (all *p* < 0.05).

### 3.3. Multinomial Logistic Regression Analysis of Biopsychosocial Predictors

A fully adjusted multinomial logistic regression analysis was performed, utilizing the three latent profiles of PR among older adults with fragility fractures as the dependent variable, with the “low resilience—physically and mentally vulnerable” profile serving as the reference group. Prespecified clinical confounders and variables significant in univariate analyses were simultaneously forced into the model. The results indicated that handgrip strength, nutritional risk, marital status, general self-efficacy, and perceived social support were significant independent predictors of PR profile membership among these patients. Additionally, specific covariates including male sex, primary education level, and comorbidity status exhibited statistical significance (Table 3).

## 4. Discussion

This study innovatively applied latent profile analysis (LPA) to examine the heterogeneity of physical resilience (PR) among older adults with fragility fractures and to clarify how distinct biopsychosocial predictors shape these profiles. By conceptualizing physical resilience not merely as a uniform static score, but as a dynamic, multidimensional capacity, our findings shift the clinical paradigm from traditional variable-centered approaches to a person-centered perspective. This provides nuanced, evidence-based support for the implementation of risk-stratified, precision-managed rehabilitative interventions.

### 4.1. Heterogeneity of Physical Resilience

Overall, the baseline PR score of the participants was 50.10 ± 8.12, indicating a moderate level of resilience. Notably, this average score is lower than that reported for older patients with chronic conditions, such as knee osteoarthritis 56.69 ± 10.53 [24]. This discrepancy highlights the fundamental differences in disease trajectories; fragility fractures act as acute stressors causing sudden homeostatic disruption, whereas knee osteoarthritis allows for gradual physiological compensation and psychological adaptation [25,26]. Building upon this overall assessment, our latent profile analysis revealed substantial underlying heterogeneity, identifying three distinct profiles: low (31.7%), moderate (51.5%), and high resilience (16.8%). This underscores that older adults do not respond uniformly to fracture stress, a reality often obscured by traditional mean-based approaches.

The “low resilience—physically and mentally vulnerable” profile accounted for nearly one-third of the participants. Patients in this category scored the lowest in the “coping and lifestyle adaptation” dimension, exhibiting pronounced vulnerability in both physiological reserve and psychological adjustment. Under the acute stress of a fracture, these patients concurrently experience severe physical impairment and low coping efficacy, making them highly susceptible to adverse psychological outcomes [27,28]. Clinical staff must identify this vulnerable population early and initiate a multidisciplinary team (MDT) intervention model to help them safely navigate this critical phase.

The “moderate resilience—limited adaptation” profile constituted the largest proportion (51.5%). These patients possess basic coping abilities but lack sufficient proactive motivation for optimal functional recovery when confronting high-intensity stress, thereby maintaining a fragile homeostatic balance. Changes in care environments post-discharge or minor complications may easily disrupt this balance, precipitating a downward shift into the low resilience profile. Nursing managers should regard this profile as the primary target for refined management. By developing individualized intervention plans targeting specific rehabilitation deficits, nurses can help these patients overcome recovery barriers.

The “high resilience—potential activated” profile comprised 16.8% of the sample. These patients maintain a robust overall PR status, objectively view their fractures, and demonstrate proactive adaptation to the negative impacts of injury and aging. They actively integrate their biopsychosocial resources to cooperate with treatment. For this group, clinical nursing should pivot toward an empowerment-based approach [29]. Nurses should respect their autonomy, guide their participation in shared decision-making for rehabilitation, and leverage peer support to build recovery confidence among other patients in the ward.

### 4.2. Biopsychosocial Predictors of Physical Resilience Profile Membership

#### 4.2.1. Physiological Reserve Factors

The multivariate logistic regression analysis revealed that decreased handgrip strength was a significant negative predictor for membership in both the moderate-resilience and high-resilience profiles relative to the low-resilience profile. This finding implies that a deficiency in muscle strength is strongly associated with a patient’s likelihood of exhibiting a state of low PR. As a core diagnostic component of sarcopenia and systemic frailty [30], handgrip strength serves as a reliable proxy for systemic muscle strength and acute stress-resistance capacity. Older adults with decreased handgrip strength often struggle to participate effectively in early bedside mobilization and rehabilitation protocols [31], which may correlate with a compounding cycle of disuse muscle atrophy and prolonged recovery trajectories.

Similarly, nutritional risk was significantly associated with an increased likelihood of being categorized into the low-resilience profile. Adequate nutrition serves as the biological foundation for tissue repair and immune regulation. The hypermetabolic state induced by acute fracture trauma and subsequent surgical stress severely depletes endogenous protein reserves. Consequently, patients facing nutritional risk may be more vulnerable due to a lower capacity to withstand this high physiological consumption [32]. These findings underscore the necessity for healthcare professionals to transition from traditional static screening approaches to dynamic, proactive physiological reserve assessments. While our cross-sectional data highlight the critical need to address concurrent muscle weakness and nutritional risks, future interventional and longitudinal studies are required to establish standardized clinical protocols. For instance, future research could explore the utility of bedside musculoskeletal ultrasound as a tool for multimodal quantitative measurements of muscle volume and quality [33]. Additionally, subsequent clinical trials are warranted to investigate proactive nutritional support, characterized by the targeted supplementation of high-quality whey protein and muscle-synthesizing nutritional substrates [34]. Ultimately, a comprehensive care model that integrates nutritional optimization with early functional training can may support the physiological reserve of older adults with fragility fractures, thereby potentially correlating with improved functional recovery outcomes and long-term quality of life.

#### 4.2.2. Psychological Factors

The multinomial logistic regression analysis demonstrated that general self-efficacy was a significant positive predictor for membership in both the moderate-resilience and high-resilience profiles relative to the low-resilience profile. This indicates that patients with elevated self-efficacy are substantially more likely to be categorized into profiles reflecting optimal PR. As a core psychological resource, self-efficacy is theoretically linked to greater patient confidence in their recuperative capabilities, which may potentially translate into better adherence to rehabilitation protocols. Clinically, patients with high self-efficacy tend to demonstrate a greater willingness to endure the physical discomfort associated with early mobilization. They may be better equipped to channel internal beliefs into proactive self-management behaviors, thereby potentially correlating with a reduced fear of movement (kinesiophobia) and fewer psychological barriers induced by acute trauma [35]. This aligns with the findings of Wang et al. [36], who reported that higher self-efficacy is significantly associated with greater PR and better functional recovery. These findings highlight the potential value of integrating a holistic biopsychosocial approach into standard orthopedic care. To build upon these cross-sectional associations, future interventional studies are needed to determine whether targeted psychological interventions—such as motivational interviewing, structured peer support, and the sharing of successful recovery narratives [37,38]—can effectively foster progressive rehabilitation empowerment. By exploring such strategies, clinical staff might better mobilize patients’ internal psychological resources, effectively supporting their transition from passive care recipients into active, engaged participants in their own recovery trajectory.

#### 4.2.3. Social and Family Support Factors

The fully adjusted multinomial logistic regression analysis revealed that having a spouse was significantly associated with membership in the high-resilience profile relative to the low-resilience profile. As primary caregivers, spouses typically provide fundamental daily assistance and emotional comfort during the acute recovery phase. This intimate support may serve as a crucial protective barrier, potentially fostering a supportive environment that is strongly linked to optimal recovery. Furthermore, alongside intimate spousal care, higher perceived social support also emerged as a significant predictor for the high-resilience profile. Comprehensive social networks—encompassing children, extended relatives, friends, and community resources—constitute a vital external buffer that complements spousal support. Consistent with previous research demonstrating the positive association between robust social support and postoperative recovery and quality of life in hip fracture patients [39], an expansive support network is theorized to alleviate psychosocial burden by providing the necessary informational, emotional, and tangible resources that are strongly linked to a state of high PR. The identification of these biopsychosocial predictors underscores the importance of optimizing discharge planning and transitional care. During clinical assessments, healthcare professionals should accurately identify vulnerable patients with deficits in these prognostic factors. Prior to discharge, clinicians could collaborate with family caregivers to seamlessly connect patients with community health services, constructing a multidimensional “hospital–community–family” safety net that aims to foster an environment conducive to functional recovery and long-term rehabilitation outcomes.

### 4.3. Clinical Implications and Study Limitations

#### 4.3.1. Clinical Implications

The findings of this study offer several pivotal implications for orthopedic and rehabilitative nursing care. First, the identified heterogeneity in PR underscores the necessity for clinicians to move away from a “one-size-fits-all” approach, instead implementing risk-stratified, targeted management based on patients’ specific latent profile memberships. Second, the prominent role of physiological reserves suggests that clinical protocols should transition from static screenings to dynamic, multimodal assessments, such as incorporating bedside musculoskeletal ultrasound to detect the “sarcopenia–malnutrition” syndrome early. Finally, the significant predictive power of self-efficacy and broad social support systems highlights the urgency of integrating structured psychological empowerment (e.g., motivational interviewing) and multidisciplinary transitional care networks into standard orthopedic pathways, ensuring a seamless “hospital–community–family” safety net for older adults navigating acute fracture stress.

#### 4.3.2. Study Limitations

Several limitations must be acknowledged. First, the multinomial logistic regression model’s stability is limited by low events per variable (EPV) and sparse data bias. Specifically, in Profile 3 (*n* = 38), extremely sparse categories like lacking a spouse (*n* = 1) or having nutritional risk (*n* = 2) produced exceptionally wide 95% confidence intervals. Although aggregating categories or applying Firth correction could artificially narrow these intervals, we deliberately retained the original categorizations and the unpenalized model to transparently preserve their real-world sociodemographic and clinical significance. Second, the cross-sectional design precludes causal inferences and cannot capture the dynamic temporal trajectories of PR. Importantly, the possibility of reverse causality cannot be entirely ruled out; for example, patients with inherently higher physical resilience may be naturally predisposed to perceive and report higher levels of self-efficacy and social support. Third, the sample was derived from a single geographical region using convenience sampling, which may limit the generalizability of our findings to diverse populations with different socioeconomic or cultural backgrounds. This sampling approach may also introduce a potential selection bias toward relatively healthier or more cooperative patients. Fourth, due to the acute post-fracture setting in which data were collected, standard baseline assessments of Activities of Daily Living (ADL) were practically challenging to obtain uniformly, and specific quantitative grading systems for fracture severity were not systematically recorded. Although we mitigated this by forcing alternative clinical proxies—such as fracture type and comorbidity burden—into the multivariable model, the potential unmeasured confounding effects of baseline ADL and precise fracture severity cannot be entirely ruled out. Future robust, multi-center longitudinal studies with larger sample sizes are warranted to yield more precise odds ratio estimates, incorporate comprehensive baseline clinical assessments, and delineate the dynamic evolutionary trajectories of physical resilience across distinct rehabilitative phases. Finally, because handgrip strength was measured in a modified semi-recumbent position due to acute fracture restrictions, applying the standard AWGS seated cut-off values might slightly underestimate actual muscle strength, potentially introducing a minor classification bias. Collectively, future robust, multi-center longitudinal studies with larger sample sizes are warranted to yield more precise estimates, incorporate comprehensive baseline clinical assessments, and delineate the dynamic evolutionary trajectories of physical resilience.

## 5. Conclusions

In conclusion, this study applied latent profile analysis to demonstrate significant psychological and physiological heterogeneity in PR among older adults with fragility fractures, successfully identifying three distinct profiles: low, moderate, and high resilience. Furthermore, multinomial logistic regression analysis underscored that PR in this population is shaped by a complex interplay of biopsychosocial predictors, where handgrip strength, nutritional risk, marital status, perceived social support, and general self-efficacy serve as critical independent determinants. These findings transition the clinical paradigm from a traditional reactive approach to a proactive, precision-managed model. To maximize functional recovery and enhance the quality of life for these vulnerable older adults, healthcare systems should prioritize the early stratification of resilience profiles and implement targeted, multidisciplinary interventions that seamlessly bridge hospital, community, and family care networks.

## Figures and Tables

**Table 1:** Fit indices of latent profile analysis models for physical resilience among older patients with fragility fractures.

Model	AIC	BIC	aBIC	Entropy	LMRT	BLRT	Proportion (%)
1	3335.484	3355.954	3336.939	—	—	—	—
2	3105.865	3139.981	3108.290	0.756	0.054	<0.001	53.4/46.6
3	**2962.895**	**3010.658**	**2966.289**	**0.865**	**<0.001**	**<0.001**	**31.7/16.8/51.5**
4	2924.563	2985.973	2928.928	0.844	0.007	<0.001	10.3/29.9/43.6/16.2
5	2910.845	2985.901	2916.180	0.810	0.207	0.013	10.3/25.9/39.3/15.2/9.3

Note: AIC = Akaike Information Criterion; BIC = Bayesian Information Criterion; aBIC = sample size-adjusted BIC; LMRT = Lo–Mendell–Rubin adjusted likelihood ratio test; BLRT = Bootstrapped likelihood ratio test. The bolded row indicates the optimal model selected.

**Table 2:** Baseline characteristics and univariate analysis of variables across three physical resilience profiles.

Characteristics	Total (*N* = 224)	Low Resilience (*n* = 70)	Moderate Resilience (*n* = 116)	High Resilience (*n* = 38)	Statistic	*p*-Value
Gender					χ^2^ = 4.088	0.129
Male	92 (41.1)	25 (27.2)	46 (50.0)	21 (22.8)		
Female	132 (58.9)	45 (34.1)	70 (53.0)	17 (12.9)		
Age					χ^2^ = 26.711	<0.001
60~69	72 (32.1)	13 (18.6)	36 (31.0)	23 (60.5)		
70~79	81 (36.2)	23 (32.9)	48 (41.4)	10 (26.3)		
≥80	71 (31.7)	34 (48.6)	32 (27.6)	5 (13.2)		
Marital status					χ^2^ = 33.826	<0.001
With spouse	168 (75.0)	36 (51.4)	95 (81.9)	37 (97.4)		
Without spouse	56 (25.0)	34 (48.6)	21 (18.1)	1 (2.6)		
Education					χ^2^ = 15.689 ^a^	0.012
Primary school or below	70 (31.3)	29 (41.4)	33 (28.4)	8 (21.1)		
Junior high school	105 (46.9)	31 (44.3)	59 (50.9)	15 (39.5)		
Senior high or technical school	39 (17.4)	9 (12.9)	21 (18.1)	9 (23.7)		
Junior college or above	10 (4.5)	1 (1.4)	3 (2.6)	6 (15.8)		
Average monthly household income					χ^2^ = 10.297	0.036
<3000	53 (23.7)	23 (32.9)	27 (23.3)	3 (7.9)		
3000~4999	107 (47.8)	29 (41.4)	59 (50.9)	19 (50.0)		
≥5000	64 (28.6)	18 (25.7)	30 (25.9)	16 (42.1)		
Handgrip strength					χ^2^ = 65.082	<0.001
Normal	104 (46.4)	8 (11.4)	62 (53.4)	34 (89.5)		
Decreased	120 (53.6)	62 (88.6)	54 (46.6)	4 (10.5)		
NRS2002					χ^2^ = 26.713	<0.001
0~2	159 (71)	35 (50.0)	88 (75.9)	36 (94.7)		
≥3	65 (29.0)	35 (50.0)	28 (24.1)	2 (5.3)		
Fracture site					χ^2^ = 22.163	0.001
Hip	88 (39.3)	41 (58.6)	35 (30.2)	12 (31.6)		
Thoracolumbar spine	67 (29.9)	16 (22.9)	43 (37.1)	8 (21.1)		
Upper extremity	39 (17.4)	7 (10.0)	20 (17.2)	12 (31.6)		
Lower extremity	30 (13.4)	6 (8.6)	18 (15.5)	6 (15.8)		
History of previous fractures					χ^2^ = 11.057	0.004
Yes	59 (26.3)	28 (40.0)	26 (22.4)	5 (13.2)		
No	165 (73.7)	42 (60.0)	90 (77.6)	33 (86.8)		
Chronic comorbidities					χ^2^ = 5.107	0.277
0	89 (40.1)	25 (35.7)	46 (40.0)	18 (48.6)		
1	101 (45.5)	30 (42.9)	55 (47.8)	16 (43.2)		
≥2	32 (14.4)	15 (21.4)	14 (12.2)	3 (8.1)		
Perceived social support [Mean ± SD]	53.57 ± 6.74	50.93 ± 5.97	53.57 ± 6.74	58.74 ± 6.22	*F* = 19.235	<0.001
General self-efficacy [Median (IQR)]	21.00 (17.00, 25.00)	18.00 (14.00, 20.00)	22.50 (17.25, 26.00)	26.00 (21.00, 30.25)	*H* = 47.607	<0.001

Note: Data are presented as *n* (%) unless specified otherwise in the variable column. IQR = Interquartile range. ^a^ Fisher’s exact test was applied due to expected frequencies < 5 in some cells.

**Table 3:** Multinomial logistic regression analysis of biopsychosocial predictors for physical resilience latent profiles.

Variables	Categories	Profile 2 vs. Profile 1				Profile 3 vs. Profile 1			
		*β*	OR	95%CI	*p*	*β*	OR	95%CI	*p*
Sex	Male	0.667	1.948	(0.838, 4.524)	0.121	1.868	6.475	(1.691, 24.800)	0.006
Marital status	With spouse	0.903	2.466	(0.946, 6.431)	0.065	2.808	16.573	(1.238, 221.933)	0.034
Education	Primary school or below	−0.796	0.451	(0.031, 6.667)	0.562	−3.725	0.024	(0.001, 0.591)	0.022
Chronic comorbidities	0	−0.529	0.589	(0.121, 2.860)	0.512	−3.317	0.036	(0.002, 0.841)	0.039
Handgrip strength	Decreased	−2.352	0.095	(0.029, 0.308)	<0.001	−3.913	0.020	(0.003, 0.120)	<0.001
NRS 2002	0–2 (No risk)	1.243	3.465	(1.085, 11.069)	0.036	3.957	52.317	(2.920, 937.424)	0.007
Perceived social support		0.027	1.027	(0.955, 1.104)	0.471	0.156	1.169	(1.046, 1.307)	0.006
General self-efficacy		0.154	1.167	(1.064, 1.280)	0.001	0.246	1.279	(1.118, 1.463)	<0.001

## Data Availability

The data supporting the findings of this study are not publicly available due to privacy and ethical restrictions, as they contain sensitive information about the study participants. The datasets generated during the current study are available from the corresponding author upon reasonable request.

## Supplementary Materials

The following supporting information can be downloaded at https://www.mdpi.com/article/10.3390/healthcare14131923/s1, Figure S1: STROBE flow diagram of the study.
