# Supplementary figures and images for: Physical Resilience and Its Influencing Factors Among Older Patients with Fragility Fractures: A Cross-Sectional Study Based on Latent Profile Analysis
Source: Healthcare (Basel). 2026 Jul 1;14(13):1923. doi: 10.3390/healthcare14131923 (PMC13362454; doi:10.3390/healthcare14131923)

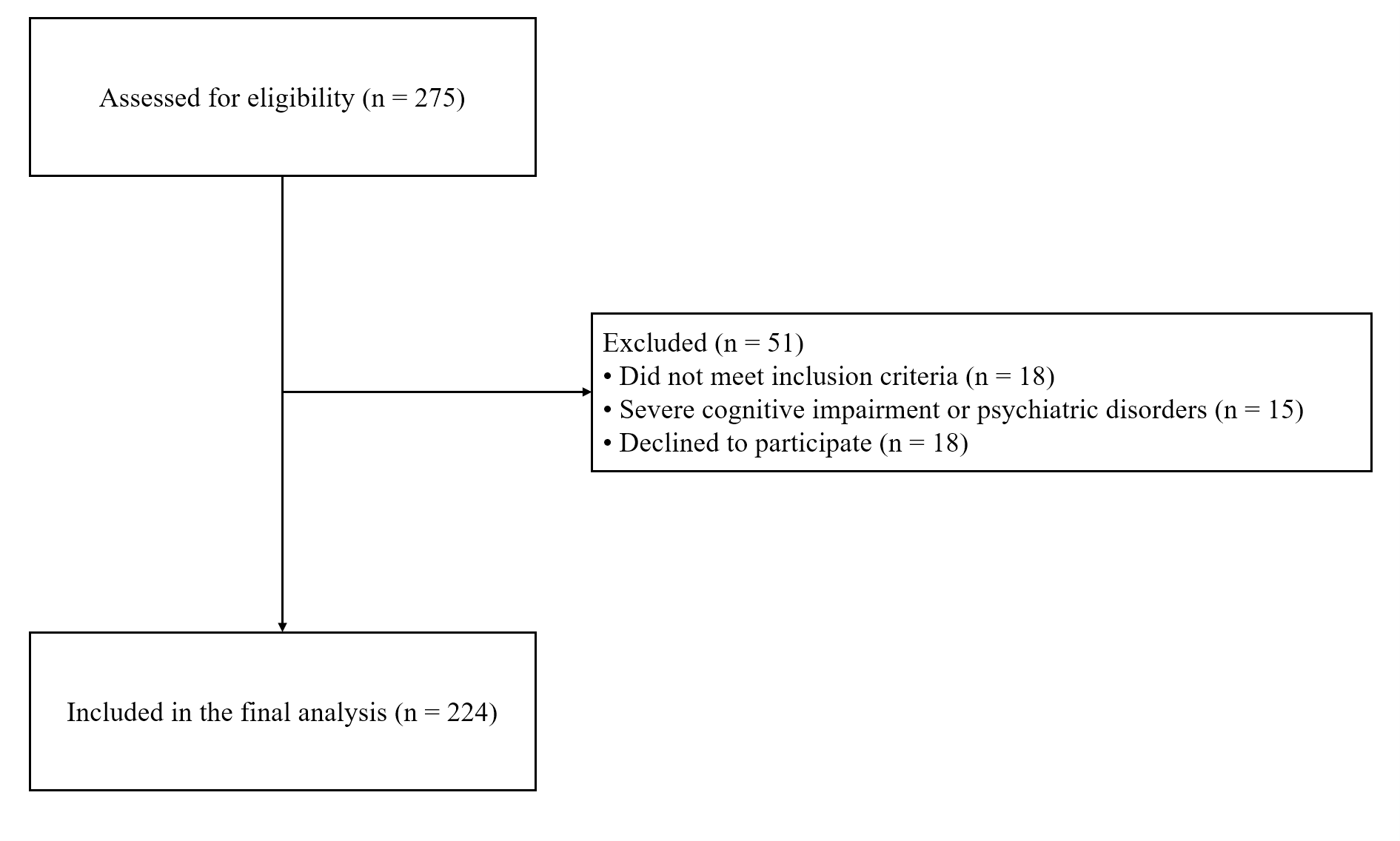

Supplement: Supplementary file 1 [file healthcare-14-01923-s001.zip › Supplementary Figure S1.tif]
